# Pharmacotherapy of Alzheimer’s Disease: Seeking Clarity in a Time of Uncertainty

**DOI:** 10.3389/fphar.2020.00261

**Published:** 2020-03-24

**Authors:** Nurul Husna Ibrahim, Mohamad Fairuz Yahaya, Wael Mohamed, Seong Lin Teoh, Chua Kien Hui, Jaya Kumar

**Affiliations:** ^1^Department of Physiology, Faculty of Medicine, Universiti Kebangsaan Malaysia Medical Centre, Kuala Lumpur, Malaysia; ^2^Department of Anatomy, Faculty of Medicine, Universiti Kebangsaan Malaysia Medical Centre, Kuala Lumpur, Malaysia; ^3^Basic Medical Science Department, Kulliyyah of Medicine, International Islamic University Malaysia, Kuantan, Malaysia; ^4^Faculty of Medicine, Department of Clinical Pharmacology, Menoufia University, Shebin El-Kom, Egypt; ^5^Glycofood Sdn Bhd, Selangor, Malaysia

**Keywords:** pharmacotherapy, Alzheimer’s disease, Alzheimer, neuroinflammation, amyloid, tau protein, glutamate

## Abstract

Alzheimer’s disease (AD) is recognized as a major health hazard that mostly affects people older than 60 years. AD is one of the biggest medical, economic, and social concerns to patients and their caregivers. AD was ranked as the 5^th^ leading cause of global deaths in 2016 by the World Health Organization (WHO). Many drugs targeting the production, aggregation, and clearance of Aβ plaques failed to give any conclusive clinical outcomes. This mainly stems from the fact that AD is not a disease attributed to a single-gene mutation. Two hallmarks of AD, Aβ plaques and neurofibrillary tangles (NFTs), can simultaneously induce other AD etiologies where every pathway is a loop of consequential events. Therefore, the focus of recent AD research has shifted to exploring other etiologies, such as neuroinflammation and central hyperexcitability. Neuroinflammation results from the hyperactivation of microglia and astrocytes that release pro-inflammatory cytokines due to the neurological insults caused by Aβ plaques and NFTs, eventually leading to synaptic dysfunction and neuronal death. This review will report the failures and side effects of many anti-Aβ drugs. In addition, emerging treatments targeting neuroinflammation in AD, such as nonsteroidal anti-inflammatory drugs (NSAIDs) and receptor-interacting serine/threonine protein kinase 1 (RIPK1), that restore calcium dyshomeostasis and microglia physiological function in clearing Aβ plaques, respectively, will be deliberately discussed. Other novel pharmacotherapy strategies in treating AD, including disease-modifying agents (DMTs), repurposing of medications used to treat non-AD illnesses, and multi target-directed ligands (MTDLs) are also reviewed. These approaches open new doors to the development of AD therapy, especially combination therapy that can cater for several targets simultaneously, hence effectively slowing or stopping AD.

## Introduction

Alzheimer's disease (AD) is a chronic neurodegenerative disease that usually affects people older than 60 years. The etiologies of many neurodegenerative diseases are not limited to a single gene or pathway, but are rather an intricate network of causatives, including neuroinflammation, oxidative stress, mitochondrial dysfunction, protein misfolding, and aggregation that can lead to cell death (Wu et al., 2018; Kamil et al., 2019). Various mechanisms were associated with the sporadic form of AD, which accounts for most AD cases, whereas mutations of three genes, including amyloid precursor protein (APP), presenilin 1 (PS1), and presenilin 2 (PS2) are heavily linked with familial AD cases (Newman et al., 2017). The irreversible symptoms of AD, such as progressive deterioration of intellect, memory, and attentiveness, are noticeable when both NFTs and Aβ plaques have disseminated through the limbic system (Canter et al., 2016).

Aβ plaques-induced brain atrophy begins with the loss of synapses and enlargement of the ventricles (Ramos-Rodriguez et al., 2017), whereas NFTs are often associated with grey matter loss (Bejanin et al., 2017). Progressive loss of cortical interneurons and specific neurotransmitter pathways such as acetylcholine (ACh), noradrenaline (NA), and serotonin (5-HT) are also reported in AD (Ribeiro et al., 2017). In the early stages of AD, typically around age 70, cells in the hippocampus start to degenerate, causing mild forgetfulness of recent events and familiar names, and also difficulty in solving simple mathematical problems (Selkoe, 2011; Bie et al., 2018). After 10 years, atrophy of the cerebral cortex occurs in moderate AD stage, resulting in a decline in language, emotional outbursts, impaired ability in conducting simple tasks such as combing hair and buttoning shirts, and an inability to think clearly. In advanced stages of AD, where more nerve cells have undergone degeneration, patients are often agitated, wandering, and unable to recognize faces and communicate (Braak and Tredici, 2018).

AD-related deaths have markedly increased over the past two decades, but a cure remains elusive (Alzheimer's Association, 2019). Treatment options for AD that are approved by the Food and Drug Administration (FDA) do not have a curative effect or are able to slow down the progression of the disease (Folch et al., 2018a). The most commonly prescribed drugs are acetylcholinesterase inhibitors (AChEIs), such as tacrine, donepezil, rivastigmine, and galantamine, and N-methyl-D-aspartate receptor (NMDAr) antagonists, such as memantine (Morsy and Trippier, 2018; **Table 1**). Tacrine was eventually discontinued due to its hepatotoxicity, and tacrine hybrids are now being studied (Sameem et al., 2017). AChEIs delay the metabolism of ACh by inhibiting acetylcholinesterase (AChE) as AD patients have a deficiency of ACh (Kumar et al., 2015). Meanwhile, memantine prevents excitotoxicity by blocking NMDAr's activation (Marttinen et al., 2018).

**Table 1:** Conventional available pharmacotherapy for Alzheimer's disease.

Drug Name	Mechanism	Dosage	References
Tacrine	AChE inhibitor	Oral q.i.d.: 10 – 20 mg (halted due to its hepatotoxicity)	(Sameem et al., 2017) (Lopes et al., 2018)
Donepezil	AChE inhibitor	Mild-to-moderate AD: Tablet q.d.: 5 mg, 10 mg Orally disintegrating tablet q,d,: 5 mg, 10 mg Moderate-to-severe AD: Tablet q.d.: 10 mg, Orally disintegrating tablet q,d,: 10 mg Severe AD: Tablet q.d.: 23 mg	(Masters et al., 2015) (Foster et al., 2016) (Zhang and Gordon, 2018) (Birks and Harvey, 2018) (Lee et al., 2015)
Rivastigmine	AChE inhibitor	Mild-to-moderate AD: Capsule b.i.d.: 1.5 mg, 3 mg, 4.5 mg, 6 mg Patch q.d.: 4.6 mg, 9.5 mg Severe AD: Patch q.d.: 13.3 mg	(Birks and Evans, 2015) (Chang et al., 2019)
Galantamine	AChE inhibitor	Mild-to-moderate AD: Tablet b.i.d.: 4 mg, 8 mg, 12 mg Solution b.i.d.: 4 mg Extended-release capsule q.d.: 8 mg, 16 mg, 24 mg	(Wake et al., 2016) (Nakayama et al., 2017) (Ohta et al., 2017) (Blautzik et al., 2016) (Oka et al., 2016)
Memantine	N-methyl-D-aspartate receptor antagonist	Moderate-to-severe AD: Tablet b.i.d.: 5 mg, 10 mg Solution b.i.d.: 2 mg/ml Extended-release capsule q.d.: 7 mg, 14 mg, 21 mg, 28 mg	(Wong et al., 2016) (Schmitt et al., 2018) (Deardorff and Grossberg, 2016) (Folch et al., 2018b) (Knight et al., 2018) (Kanasty et al., 2019)

q.i.d., four times a day; q.d., once a day; b.i.d., twice a day.

Heterogeneity in AD pathogenesis impedes the development of curative strategies. Three neuropathological mechanisms postulated in AD are: i) formation of extracellular Aβ plaques by insoluble amyloid proteins' aggregate, ii) formation of NFTs (disorganized bundles of filaments in the neuronal cytoplasm) by hyperphosphorylated tau proteins, and iii) neuronal loss as the aftermath of the Aβ plaques and NFTs (Revett et al., 2013). Thus, many drugs failed to improve cognition in mild-to-moderate AD patients as the drugs target a single pathology without acknowledging other neurological insults (Jobke et al., 2018). Hence, a multi-target approach is being highly investigated in clinical trials to synergistically target distinct pathways and ameliorate AD (Cummings et al., 2019).

## Amyloid Plaques

Overproduction and reduced clearance of Aβ_42_ monomers cause the deposition of Aβ plaques that eventually leads to alteration in downstream neurobiological events (Takahashi et al., 2017). Aβ plaques cause catastrophic damage to cellular membranes' integrity through the formation of the membrane's pore and the reduction of the membrane's fluidity, hence, leading to neuronal death (Yasumoto et al., 2019). Aβ plaques also trigger the activation of microglia and astrocyte as an inflammatory response, alter the neuronal calcium homeostasis causing oxidative injury, and disrupt the protein kinase and phosphatase-related pathways, resulting in hyperphosphorylation of tau and formation of NFTs (Reiman, 2016). Furthermore, self-propagation of Aβ_42_ plaques and hyperphosphorylated tau, *via* a prion-like mechanism, may exaggerate the synaptic dysfunction, neurotransmitter deficits, and neuronal loss in the brain (Goedert, 2015).

Although Aβ plaques alone may not be adequate in causing the transmission of pathological tau, amyloid cascade hypothesis suggests that deposition Aβ plaques is the triggering factor for the cognitive deteriorations in AD (Blennow et al., 2015). Hence, future drug development should seek to determine whether a single-target therapy targeting Aβ is sufficient to treat AD or whether a combination therapy between anti-Aβ and anti-tau is needed (He et al., 2018).

## Neurofibrillary Tangles

Intracellular NFTs are the deposits of insoluble proteins in neuronal cell bodies (Vanden Dries et al., 2017). Tau is a cytoskeletal microtubule-associated protein (MAP) that is phosphorylated at three sites - serine (S), threonine (T), and at residues adjacent to proline - and binds at the microtubules (MTs) to sustain the MTs' stability and integrity (Pradeepkiran et al., 2019). The toxicity of tau can impair neuronal function depending on its post-translational modifications. The most potent phosphorylations of tau take place at T231, S235, and S262, which results in the loss of tau's ability to bind to MTs, leading to tau self-assembly into paired helical filaments (PHF) (Iqbal et al., 2018).

Phosphorylation of tau detaches it from MTs to allow the intracellular transportation of subcellular organelles such as mitochondria and lysosomes from the nerve terminals to the cells' soma through secretory vesicles (Pradeepkiran et al., 2019). Hyperphosphorylation of tau sequesters the normal tau in which it may excessively impair tau binding and destabilize MTs, thus, impairing the axonal transport causing neurodegeneration through synaptic starvation, neurite outgrowth, and neuronal death (Minjarez et al., 2013). Hyperphosphorylated tau tends to misfold and forms PHF which eventually aggregates to form NFTs as a defense mechanism in the cell soma (Gandini et al., 2018).

In contrast to Aβ pathology, which causes hyperactivity of neurons, tau silences the neurons (Busche et al., 2019). This provokes the question on how the coexistence of Aβ and tau pathologies causes neurodegeneration in AD. From the fully eradicated neuronal hyperactivity and drastic decline of cortical activity in rats with both Aβ and tau pathologies, it can be concluded that deposition of Aβ plaques may be the triggering factor that sparks other AD etiologies, but tau pathology is the one dominating the aftermath effects of this dual proteinopathies in AD. It is tau pathology that determines the cognitive status in AD compared to Aβ pathology, which is another solid reason for the constant failures of Aβ drugs. The combination of anti-amyloid and anti-tau is crucial, as suppressing gene expression of tau is less effective in restoring the neuronal impairments in the presence of Aβ plaques (DeVos et al., 2018).

## Current Drugs Targeting Aβ - Failures

According to the updated AD drug development pipeline in 2018, although more than 50% of drugs in Phase III trials are targeting Aβ, there is still a steep 40% decline from year 2017 to 2018 in anti-Aβ drugs in Phase I and II trials, which manifests the shift in AD research following the repetitive failures of anti-Aβ drugs (Mullane and Williams, 2018) (**Table 2**). Reducing the generation of Aβ_42_, inhibiting the aggregation of Aβ plaques, or increasing the rate of Aβ clearance from the cerebrospinal fluid (CSF) and brain are the common approaches of anti-Aβ drugs (Scheltens et al., 2016). At present, the complexity of AD's pathogenesis is vaguely understood, which may involve numerous other proteins beside Aβ and various biological pathways (Doig et al., 2017). This multifactorial AD pathogenesis is most probably the main reason for the repetitive failures of anti-amyloid drugs because a single target treatment may not be able to cater for all the altered pathways involved in the neurodegenerative events (Selkoe, 2019).

**Table 2:** Failed clinical trials of anti-Aβ drugs for the treatments of Alzheimer's disease.

Name	Therapy Type	Clinical Trials	Cohort	Reason of failure	References
Solanezumab	IgG1 humanized anti-Aβ mAbs	III	Mild-to-moderate AD	Lack of efficacy	(Honig et al., 2018)
Bapineurumab	IgG1 humanized anti-Aβ mAbs	III	Mild-to-moderate AD	Lack of efficacy	(Salloway et al., 2018) (Ketter et al., 2017)
Crenezumab	IgG1 humanized anti-Aβ mAbs	II	Mild-to-moderate AD	Lack of efficacy, Did not meet primary and secondary endpoint.	(Cummings et al., 2018)
Gantenerumab	IgG1 humanized anti-Aβ mAbs	III	Prodromal AD	Halted due to futility, no significant differences observed in primary and secondary endpoint.	(Ostrowitzki et al., 2017)
Aducanumab	IgG1 humanized anti-Aβ mAbs	III	Mild-to-moderate AD	Lack of efficacy	(Selkoe, 2019) (Haeberlein et al., 2018) (Sevigny et al., 2016)
Tramiprosate	Aβ aggregation inhibitor	II		Lack of efficacy	(Selkoe, 2011) (Sabbagh, 2017) (Kocis et al., 2017) (Malouf and Collins, 2018)
Semagacestat	γ-secretase inhibitor	III		Lack of efficacy, Worsens cognition function at higher doses, High incidence of skin cancer and infections	(Doody et al., 2013) (Henley et al., 2014)
Verubecestat	BACE1 inhibitor	III	Mild-to-moderate AD Prodormal AD	Lack of efficacy, Adverse events: Occurrence of rash Changes in hair color Tend to have more falls and injuries Weight loss Sleep disturbance Suicidal ideation	(Egan et al., 2018) (Kennedy et al., 2016)
Lanabecestat	BACE1 inhibitor	III	Early AD Mild-to-moderate AD	Unlikely to meet primary endpoint, stopped for futility	(Eli Lilly and Company, 2018) (Cebers et al., 2017) (Eketjall et al., 2016)
Atabecestat	BACE1 inhibitor	II/III	Early AD	Adverse events: Elevation of liver enzymes	(Timmers et al., 2018) (Taylor, 2018) (Janssen, 2018)
Avagacestat	γ-secretase inhibitor	II	Prodromal AD	Lack of efficacy Adverse events: Weight loss Glycosuria	(Coric et al., 2015)

Initially, these anti-Aβ agents show potential curative effects through effective Aβ clearance in CSF and the brain during the early stage of drug development. However, the Aβ level in mild-to-moderate AD patients or even prodromal AD patients may have reached the threshold of irreversible neurotoxicity, which is the main reason for the failure of many anti-Aβ drugs once proceeded to phase III trials (Dobrowolska Zakaria and Vassar, 2018). This is probably due to lack of AD biomarkers in the past to ensure early detection and recruitment of potential AD patients for clinical trials (Folch et al., 2018b). Plus, Aβ plaques accumulate at a slow rate which may provide a large time window for a potential intervention that could either enhance the clearance or hinder the accumulation of Aβ insoluble proteins before brain atrophy and memory impairment commences (Villemagne et al., 2013). According to PET images taken by Vlassenko and colleagues, significant changes in CSF Aβ level and Aβ deposition in the brain of AD patients were reported, respectively 25 and 15 years prior to the clinical representation of AD symptoms (Vlassenko et al., 2012). This finding suggested an optimal time for early intervention of AD; hence, early treatment can be given to the presymptomatic AD patients who should have been recruited for clinical trials rather than symptomatic AD patients. This is mainly because symptomatic AD patients usually have irreversible synaptic loss and neuronal deaths. Therefore, better recruitment of patients with earlier stage of neurodegeneration and more consistent pathology underlying their AD to participate in clinical trials may generate more beneficial clinical outcomes (Briggs et al., 2016).

The targeted protein to minimize the generation of Aβ42 is β-secretase 1 (BACE1), which is involved in the first proteolytic cleavage of the APP protein and also γ-secretase which plays a role in the second cleavage in order to produce the Aβ42 protein.Verubecestat, a BACE1 inhibitor, was discontinued from the phase III trial due to its lack of efficacy and inability to establish a positive risk/benefit ratio towards mild-to-moderate and prodromal AD patients, although significant reduction of Aβ in the patients' CSF and brains were achieved during the trial (Egan et al., 2018). This finding emphasized that solely targeting amyloid may not be an appropriate strategy in treating AD. Lanabecestat is a selective BACE1 inhibitor with satisfying blood-brain barrier (BBB) penetration, high potency and permeability, and slow off-rate that is critical for its efficacy (Eketjall et al., 2016). However, lanabecestat was discontinued from the trials due to its unlikeliness to meet the primary end points in mild-to-moderate and prodromal AD patients based on a recommendation from the data monitoring committee (Panza et al., 2019). Meanwhile, a phase II/III trial of a non-selective BACE1, atabecestat, was halted as the benefit/risk ratio was no longer favorable due to chronic elevations of liver enzymes observed during the trial (Taylor, 2018).

The clinical trial of semagacestat was rushed without a strong foundation of knowledge on the compound's physiological, structural, and functional properties (De Strooper, 2014). It was even more perplexing when the trial of semagacestat continued to the next phase without any significant results from the previous trial and was terminated before the completion of phase III trial. The drug was not even as effective as the placebo, aggravated cognitive deterioration at higher doses, and demonstrated high incidence of skin cancer and infections in the study group (Doody et al., 2013). The drug's adverse effects may potentially be linked to the altered Notch signaling pathway, where Notch was one of the alternate substrates for γ-secretase, important for cell differentiation. Blocking γ-secretase through semagacestat may have blocked the differentiation of cells vital for the immune system, pigmentation, and gastrointestinal functions such as B and T lymphocytes, melanocyte stem cells, and gastrointestinal epithelial cells (Henley et al., 2014). The loss of Notch signaling by semagacestat may trigger mutations responsible for the development of skin cancer in patients' groups receiving semagacestat (Nowell and Radtke, 2017).

Apart from that, tramiprosate was an anti-glycosaminoglycan compound that targeted the inhibition of Aβ aggregation studied until the phase II trial (Folch et al., 2018b). Aβ binds with glycosaminoglycan on the cell surface for cellular uptake and internalization depending on the electrostatic interaction between the positively charged Aβ and negatively charged sulfate residue on the glycoaminoglycan (Stopschinski et al., 2018). Tramiprosate was also withdrawn from the trial due to a lack of consistent cognitive improvement as measured in Alzheimer's Disease Assessment Scale-cognitive subscale (ADAS-Cog) (Aisen et al., 2011). Nevertheless, tramiprosate was found to have higher efficacy to apolipoprotein E4 homozygotes in AD patients. Therefore, a thorough molecular analysis elucidated the mechanism of action of tramiprosate in aggregate Aβ_42_ that may be advantageous for further development of tramiprosate as an AD therapeutic regimen (Sabbagh, 2017). Concisely, tramiprosate prevents the misfolding of self-assembled Aβ_42_ monomers by enveloping them, thereby hindering the aggregation of neurotoxic Aβ_42_ plaques (Kocis et al., 2017).

Besides, extensive development of Aβ humanized IgGI monoclonal antibodies agents, such as solanezumab and bapineuzumab, that targeted the central epitopes of soluble Aβ monomers and the N-terminus of Aβ_42_ were also halted due to a lack of efficacy in mild-to-moderate AD patients (Honig et al., 2018; Castellani et al., 2019). After repetitive failures in phase III clinical trials in mild-to-moderate AD patients, EXPEDITION 1, EXPEDITION 2 and EXPEDITION 3, solanezumab are currently being tested in asymptomatic and mildly symptomatic patients with biomarker evidence of Aβ plaques deposition in brains as a preventive strategy towards AD (Willis et al., 2018). A combined therapy of solanezumab and gantenerumab was also terminated due to their lack of clinical advantages and apparent side effects when combined with BACE1 inhibitor (Cummings et al., 2019). The combination therapy was initiated to enhance the immune response towards Aβ plaques, hence, promoting Aβ clearance while BACE1 inhibits the generation of new Aβ (Folch et al., 2018b). Nonetheless, there are two emerging anti-Aβ oligomers monoclonal antibodies with promising efficacy, aducanumab and BAN2401, that bind to insoluble fibrils and soluble Aβ protofibrils, thus, relieving the brains' Aβ burden with positive impact on cognition (Panza et al., 2019). Aducanumab is an Aβ-targeting monoclonal antibody that is currently showing significant dose-dependent reduction of Aβ plaques' size (Haeberlein et al., 2018). Besides binding to both forms of Aβ, soluble oligomers and insoluble fibrils, aducanumab also alleviates calcium dyshomeostasis in affected neurons, since neuronal calcium was found altered in AD brains (Gamage and Kumar, 2017). Further studies should investigate whether the alleviation of elevated intracellular calcium by aducanumab plays a role in restoring the cognitive functions in AD. In a phase III trial, aducanumab failed to slow down cognitive deterioration in mild-to-moderate patients due to reasons such as the patients were irreversibly symptomatic, targeting Aβ was not sufficient as it may have already caused irreversible synapse and microglia toxicity, and multifactorial AD may require combination therapy (Selkoe, 2019).

BAN2401 is a humanized monoclonal antibody with encouraging therapeutic effects in treating AD. The drug is highly selective to Aβ protofibrils and recedes the formation of Aβ plaques, causing a 30% delay to cognitive impairment in mild-to-moderate AD patients within 18 months and a 47% delay by the highest dose in a phase II trial (Swanson et al., 2018). However, future studies should explore the potential of this drug in a larger group (Mendes and Palmer, 2018; Panza et al., 2019).

Passive immunization is the most predominant therapeutic approach in targeting Aβ where exogenous monoclonal antibodies (mAbs) are administered to the patients. However, this approach has been weighted with repetitive failures that subsequently theorized several augmentations for the anti-Aβ mAbs development (Piton et al., 2018). Firstly, mAbs targeting the N-terminus of Aβ are highly potent in suppressing the aggregation of Aβ and disaggregating the pre-existing Aβ fibrils (van Dyck, 2018). Next, better penetration through the BBB and higher doses of mAbs may be tested in the future since mAbs have astonishing safety profiles. Finally, recruiting preclinical AD patients for the prevention of AD clinical trials is one of the initiatives taken to maximize the benefits of anti-Aβ mAbs in treating AD at an early stage (Hampel et al., 2010).

## Emerging Treatments: Neuroinflammation

**Figure 1:**
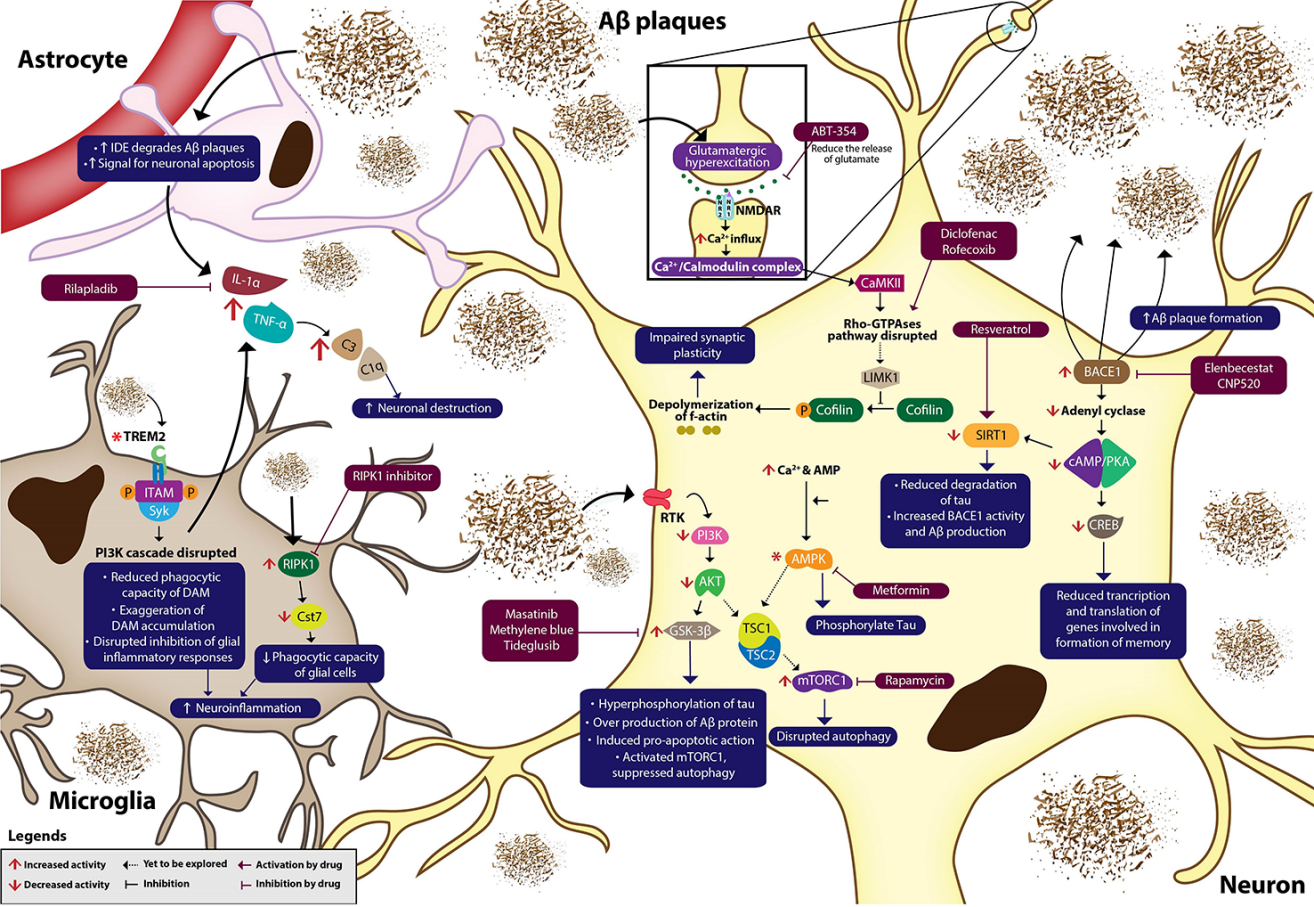
Neuroinflammation in Alzheimer's disease. Aβ plaques, NFTs and oxidative stress dysregulate various signaling cascades, causing neuroinflammation, and eventually neurodegeneration. Multiple novel pharmacotherapies ameliorate AD by normalizing the dysregulated signaling pathways in AD. IDE, insulin-degrading enzyme; Aβ, amyloid βeta; IL-1α, interleukin 1α; TNF-α, tumour necrosis factor-α; C3, complement component 3; C1q, complement protein 1q; TREM2, triggering receptor expressed on myeloid cells 2; ITAM, immunoreceptor tyrosine-based activation motif; SYK, spleen tyrosine kinase; P, phosphate; PI3K, phosphatidylinositol 3-kinase; NFκB, nuclear factor kappa β; RIPK1, receptor-interacting serine/threonine-protein kinase 1; *Cst7*, cystatin F gene; RTK, receptor tyrosine kinase; PDK1, phosphoinositide-dependent kinase 1; mTOR, mammalian Target of Rapamycin; Akt, protein kinase B; GSK-3β, glycogen synthase kinase 3β; TSC, tuberous sclerosis complex; AMP, adenosine monophosphate; AMPK, AMP-activated protein kinase; SIRT1, silent information regulator type 1; BACE1, β-secretase 1; cAMP, cyclic adenosine monophosphate; PKA, protein kinase A; CREB, cAMP response element binding protein; NMDAR, NMDA receptor; Ca²⁺/Calmodulin-dependent protein kinase II (CAMKII); LIMK1, LIM kinase 1; BDNF, brain-derived neurotrophic factor. Asterisk (*) in the diagram indicates uncertain changes of activity in AD.

Recently, the drive for new therapeutic strategies has focused on neuroinflammation interceded by microglia and astrocytes in AD pathogenesis, rather than the accustomed AD hypotheses such as Aβ and tau pathologies. This has resulted in extensive investigations of anti-inflammatory and antioxidant agents (Heneka et al., 2015). In a healthy brain, microglia provide protection against exogenous insult, while astrocytes furnish nutritional and structural support for neurons (Van Eldik et al., 2016). At the early stages of AD, excessive deposition of extracellular Aβ plaques and continuous activation of glial cells cause the release of inflammatory cytokines such as interleukin-1α (IL-1α), tumor necrosis factor-α (TNF-α), and complement protein (C1q) (**Figure 1**). The cytokines also increase astrocytes' expression of insulin-degrading enzyme (IDE) for Aβ degradation and signal for neuronal apoptosis (Son et al., 2016). NFκB signaling cascades enhanced in glial cells of AD brain to produce various inflammatory and immune proteins, including compliment component 3 (C3) that initiates neural destruction through complement-mediated synapse pruning when it binds to C3a receptors on a neuron (Benarroch, 2018).

Constant production of inflammatory cytokines by microglia leads to neuroinflammation and synaptic loss. Neuroinflammation suppresses the phagocytosis of Aβ plaques which may aggravate neurodegeneration (Cianciulli et al., 2020). Despite the undefined mechanism of rilapladib, it is presumed that rilapladib reduces neuroinflammation through the reduction of proinflammatory cytokines and restoration of BBB integrity (Maher-Edwards et al., 2015; Huang et al., 2020).

Microglia are activated into disease-associated microglia (DAM) through triggering receptors expressed on myeloid cells 2 (TREM2) independent and dependent pathways during the microglia-Aβ plaques interaction, which facilitate Aβ plaques phagocytosis and suppresses overproduction of inflammatory cytokines (Keren-Shaul et al., 2017). Activation of TREM2 leads to the phosphorylation of immunoreceptor tyrosine-based activation motif (ITAM) that causes the spleen tyrosine kinase (SYK) to dock within the receptor complex and activates the phosphatidylinositol 3-kinase (PI3K) cascades (Zheng et al., 2018). The initial response following the activation of PI3K pathway was to produce pro-inflammatory cytokines as a neuroprotective feedback (Cianciulli et al., 2016). Several studies reported that mutations of TREM2 in AD reduced the phagocytic capacity of DAM, disrupted the downstream PI3K pathway, and impaired suppression of pro-inflammatory cytokines released by the glial cells (Jay et al., 2017; Achebe et al., 2018). Meanwhile, overexpression of TREM2 ameliorated neuroinflammation by inhibiting the pro-inflammatory responses initiated by microglia (Ren et al., 2018).

In addition, restoring the microglia function in Aβ clearance may open new doors for the development of AD treatments through inhibiting receptor-interacting serine/threonine protein kinase 1 (RIPK1) that are highly expressed in microglia of AD brains (Mullard, 2018). Inhibition of RIPK1 reduces the overexpression of *Cst7* that encodes for cystatin F, an endosomal/lysosomal cathepsin inhibitor, which reduces the phagocytic capacity of primary immune cells (Ofengeim et al., 2017). Therefore, RIPK1 inhibitor is suggested to encounter neuroinflammation caused by inflamed microglia that disrupts the phagocytosis of toxic dead cells by reducing the expression of *Cst 7* and escalating Aβ plaques clearance.

Overstimulation of glutamate receptors leads to the progression of many CNS-related complications (Kumar et al., 2018a; Kumar et al., 2018b). Aβ plaques cause glutamatergic hyperexcitation through continuous stimulation of NMDAr that results in its desensitization and an increase in Ca^2+^ influx (Wang X. P. et al., 2019). NSAIDs such as diclofenac and rofecoxib were said to be a potential strategy in combating neuroinflammation by regulating Ca^2+^ homeostasis, tau phosphorylation, axonal growth, and astrocyte motility through Rho-GTPases pathway (Kumar et al., 2015). The binding of Ca^2+^ with calmodulin forms Ca^2+^/Calmodulin complex, which subsequently activates Ca²⁺/Calmodulin-dependent protein kinase II (CAMKII) and Rho-GTPases pathway, which is important for in spine morphogenesis during the induction of long-term potentiation (LTP) (Luo et al., 2016). LIM kinase 1 (LIMK1), a downstream kinase in Rho-GTPases pathway, phosphorylates and inhibits cofilin, a protein involved in the depolymerization of f-actin (Fan et al., 2018). Aβ was reported to disrupt the Rho-GTPases pathway, which regulates the dynamic of polymerization and depolymerization of f-actin to maintain the neurons' morphology (Ferrera et al., 2017). Meanwhile, ibuprofen was shown to phosphorylate cofilin at S3 to inhibit cofilin, thus, preventing the depolymerization of f-actin and impairment of synaptic plasticity. Besides, NSAIDs also suppress the microglial activation and lessen the accumulation of activated microglia (O'Bryant et al., 2018). But, NSAIDs tend to cause toxicity due to its non-selective activity. In addition, rofecoxib, a specific cyclooxygenase-2 (COX-2) inhibitor was studied in AD as COX-2 mRNAs were upregulated in AD brains (Wang et al., 2015). Inhibiting COX-2 may also hinder the decline of LTP provoked by the deposition of Aβ in the hippocampus (Deardorff and Grossberg, 2017).

Besides, phenolic compounds with antioxidant properties such as oleuropein and epigallocatechin gallate (EGCG) are also of interest for early intervention in AD management (Kamil et al., 2018). This is mainly because a significant deficiency in brain antioxidant levels was identified as the oxidative stress marker in AD (Sharman et al., 2019). For instance, EGCG was said to suppress the Aβ plaque-induced upregulation of proinflammatory cytokines in microglia, and also upregulate the expression of endogenous antioxidants such as nuclear arythroid-2 related factor 2 (Nrf2) and heme oxygenase-1 (HO-1). Hence, EGCG elicits a protective effect against oxidative stress and neuroinflammation (Cheng-Chung Wei et al., 2016).

## Novel Pharmacology: Molecular Targets

Development of novel AD pharmacotherapy is becoming profoundly important as the complexity of AD pathogenesis becomes better understood in recent times, resulting in the exploration of multitude targets in AD therapeutic strategies (**Table 3**). The pipeline of AD treatments is augmented with compounds that either modify the underlying AD pathophysiology, target several molecular targets synergistically, or repurposed as an anti-Alzheimer's drug (Bachurin etal., 2017).

**Table 3:** Novel clinical trials for Alzheimer's disease.

Name	Mechanism	Clinical trials	References
Masatinib (AB1010)	GSK-3β Inhibitor Tyrosine kinase inhibitor target mast cells and macrophages.	Phase II/III trial on mild-to-moderate AD patients Escalated dose: 4.5 mg/kg/day b.i.d., escalate to 6 mg/kg/day after 3 months' treatment Fixed dose: 4.5 mg/kg/day b.i.d. and 3.0 mg/kg/day b.i.d. Primary endpoint: ADCS-ADL that indicates self-care and activities of daily living ADAS-Cog that measure the effect on cognition and memory Secondary endpoint: MMSE CIBIC-plus	(Palomo et al., 2017) (AB Science SA, 2019) (Folch et al., 2015)
Methylene blue (MB) NCT02380573	Inhibit the formation of tau oligomers	Phase II clinical trial on healthy aging, mild cognitive impairment (MCI), and mild AD patients Primary endpoint: Working memory task Working memory task response Episodic memory task Episodic memory response Sustained attention task Neurological battery composite score Secondary endpoint: Cerebral blood flow measures	(Cummings et al., 2019) (Soeda et al., 2019) (Gauthier et al., 2016)
Metformin NCT01965756	Biguanide class medication Decrease insulin level that affect the clearance of Aβ in brain Decrease advanced glycation end products and inflammation in AD	Phase II clinical trial on MCI and early AD patients Metformin > Placebo oral metformin for 8 weeks (500 mg q.d. for 1 week, increased dose by 500 mg per week until a maximum dose of 2000 mg per day), followed by 8 weeks of placebo Placebo > Metformin After 8 weeks of placebo, oral metformin for 8 weeks (500 mg q.d. for 1 week, increased dose by 500 mg per week until a maximum dose of 2000 mg per day) Primary endpoint: Word List Memory Total (ADAS-cog) Secondary endpoint: Trails-B	(Ou et al., 2018) (Luchsinger et al., 2016) (Campbell et al., 2017) (Weinstein et al., 2019)
RPEL	Improve inhibition of AChE, reduce Aβ aggregation and reduce phosphorylation of tau	*In vivo* and *in vitro* studies	(Sergeant et al., 2019)
Tideglusib NCT01350362	Thiadiazolidinone acts as an GSK-3β inhibitor, reduce tau phosphorylation and prevent neurons apoptosis. Anti-inflammatory	Phase II clinical trial on mild-to-moderate AD patients for 26 weeks Oral tideglusib 1000 mg q.d. Oral tideglusib 1000 mg.q.o.d. once every other day Oral tideglusib 500 mg q.d. Placebo q.d. Primary endpoint: ADAS-Cog+	(Del Ser et al., 2013) (Wang and Mandelkow, 2016)
Elenbecestat NCT03036280	BACE1 inhibitor that inhibit BACE1 involved in amyloid precursor protein (APP) proteolytic cleavage during the formation of Aβ	Phase II clinical trial on prodromal AD and mild-to-moderate AD patients Phase III clinical trial on early AD patients Dosage: 50 mg q.d in the morning MissionAD1 and MissionAD2 - Phase III trial on early AD with positive biomarkers for brain amyloid pathology. Primary endpoint: CDR-SB Contact dermatitis, upper respiratory infection, headache, diarrhea, fall and dermatitis.	(Folch et al., 2018b) (Panza et al., 2018)
BAN2401 NCT03887455	IgG1 humanized anti-Aβ mAbs that binds selectively to Aβ protofibrils.	Phase III clinical trial on early AD patients Dosage: 10 mg/kg i.v. BAN2401 biweekly Primary endpoint: Change on CDR-SB from baseline Number of participants with treatment-emergent adverse events (TEAEs) Secondary endpoint: Change on amyloid Positron Emission Tomography (PET) from baseline Change on ADCOMS from baseline Change on ADAS-cog 14 from baseline	(BioArctic AB and Eisai Co., 2019) (Swanson et al., 2018) (Logovinsky et al., 2016)
CT1812 NCT03522129	Lipophilic isoindoline that bind allosterically to sigma-2 receptor complex and destabilize the Aβ oligomers binding at synapses' neuronal receptors.	Phase I clinical trial on mild-to-moderate AD patients Dosage: 90 mg, 280 mg, 560 mg CT1812 Primary endpoint: Displacement of Aβ oligomers into CSF	(Grundman et al., 2019) (Catalano et al., 2017)
Nilotinib NCT02947893	Inhibit brain Aβ, Decrease Aβ and pTau Modulate brain and peripheral immune profiles Reverse cognitive decline in AD	Phase II clinical trial on mild-to-moderate AD patients Dosage: oral 150 mg/capsule nilotinib q.d, 2 capsules after 6 months of 1 capsule Primary endpoint: Number of participants with adverse events/abnormal laboratory values	(Weinstein, 2018) (Pagan et al., 2016) (Nishioka et al., 2016)
Acitretin NCT01078168	α-secretase enhancer/amyloid aggregation inhibitor, Retinoic acid receptor agonist	Phase II clinical trial on mild-to-moderate AD patients Dosage: oral 30 mg q.d. Primary endpoint: Difference in soluble alpha-cleaved APP in CSF	(dos Santos Guilherme et al., 2019) (Freese et al., 2014)
Pinitol(NIC5-15) NCT00470418	α-secretase inhibitor that is Notch sparing	Phase II clinical trial on AD patients Primary endpoint: Number of participants with adverse events	(Anandakumar et al., 2018) (López-Sánchez et al, 2018)
Bryostatin NCT02431468	α-secretase enhancer, PKC modulator – immunomodulatory effect, increase cognitive ability	Phase II clinical trial on moderately severe-to-severe AD patients Dosage: 20 & 40 μg Bryostatin, i.v. Primary endpoint: Number of participants with TEAE and SAE Change in Severe Impairment Battery (SIB) in the Full Analysis Set (FAS) Secondary endpoint: SIB ADCS-ADL-SEV MMSE-2 NPI CGI-I	(Farlow et al., 2019)
Bexarotene NCT01782742	Retinoid X receptors (RXR) agonist to reduce Aβ in the brain	Phase II clinical trial on mild-to-moderate AD patients Dosage: 75 mg of bexarotene b.i.d., 150 mg after 1 week Primary endpoint: Change in brain amyloid burden measured by standard uptake unit regional (SUVr) according to genotypes involved in this trial Secondary endpoint: MMSE ADAS-cog CDR NPI ADCS-ADL Serum Aβ_1-42_ level Ratio of Aβ_42_ to Aβ_40_ in non-ApoE4 carriers	(Cummings et al., 2016)
ELND005 (formerly known as AZD-103), scyllo-inositol NCT01735630	Inhibit the build-up of amyloid protein in AD brains	Phase II clinical trial on moderate-to-severe AD patients Dosage: ELND005 tablets, b.i.d. for 12 weeks Primary endpoint: Change in NPI-C combined agitation and aggression Secondary endpoint: ADCS-CGIC NPI MMSE ADCS-ADL	(Lee et al., 2017)
ABT-354 NCT01908010	5-HT_6_ antagonist regulate the release of acetylcholine, glutamate and noradrenaline in the forebrain region.	Phase I clinical trial on mild-to-moderate AD patients Primary endpoint: Vital signs ECG Neurological exam Laboratory tests (hematology, chemistry, urinalysis) Number of participants with adverse events C-SSRS Secondary endpoint Pharmacokinetic parameters	(Ferrera et al., 2017) (Lalut et al., 2017)
CNP520 NCT03131453	BACE1 Inhibitor	Generation Study 2 – Phase II/III trial on homozygotes *APOE ϵ4* and heterozygotes *APOE ϵ4* carriers with elevated brain amyloid. Dosage: p.o. 15 mg/day or 50 mg/day CNP520 Primary endpoint: diagnosis of MCI APCC test score.	(Lopez et al., 2019) (Panza et al., 2018) (Borowsky et al., 2019)
Crenezumab NCT02670083	Amyloid monoclonal antibodies	Phase III clinical trial on prodromal to lid AD patients Dosage: i.v. crenezumab q4w for 100 weeks Primary endpoint: Change in CDR-SB	(Cummings et al., 2018)
Rilapladib NCT01428453	Lipoprotein-associated phospholipase A_2_ (Lp-PLA_2_) inhibitor that suppress neuroinflammation	Phase IIa clinical trial on AD patients Dosage: 250 mg rilapladib Primary endpoint: Change in Aβ_42_ and Aβ_40_ level in CSF Ratio of Aβ_42_/Aβ_40_ in CSF Tau and p-tau in CSF Working memory/executive function (WM/EF) composite score	(Maher-Edwards et al., 2015)
Edonerpic Maleate (T-817MA) NCT02079909	Activate sigma-1 receptor and regulate the microglial function.	Phase II clinical trial on mild-to-moderate AD patients Dosage: 224 mg of T-817MA q.d., 448 mg after 4 weeks Primary endpoint: Change in ADAS-cog CGIC Secondary endpoint: ADCS-ADL	(Schneider et al., 2019)
Carvedilol NCT01354444	Non-selective B-adrenergic receptor blocker that indirectly reduce neurons' apoptosis.	Phase IV clinical trial on AD patients Dosage: 25 mg of carvediol daily Primary endpoint: Hopkins Verball Learning Test (HVLT) Secondary endpoint: Aβ oligomers level in CSF	(Liu and Wang, 2018)
Intepirdine (RVT-101) NCT02585934	5-HT_6_ antagonist	Phase III clinical trial on AD patients Dosage: 35 mg of oral RVT-101 q.d. Primary endpoint: Change in ADAS-cog 11 ADCS-ADL Secondary endpoint: CIBIC+ Dependence Scale (DS) Neuropsychiatric Inventory (NPI) ADAS-cog 13 Plasma concentration of RVT-101	(Lombardo et al., 2017a) (Lombardo et al., 2017b) (Zhu et al., 2017)
Vanutide Cridificar (ACC-001) NCT00479557	Vaccine that produce Aβ-directed B-cell response.	Phase II clinical trial on mild-to-moderate AD patients Dosage: 3, 10, 30 μg IM on day 1, month 1, 3,6, and 12 Primary endpoint: Percentage of participants with treatment-emergent adverse events (TEAEs) or serious adverse events (SAE) Secondary endpoint: Geometric mean titers (GMTs) of Anti-Aβ immunoglobulin (IgG) using Enzyme-linked Immunosorbent Assay (ELISA)	(Pasquier et al., 2016)
Resveratrol NCT01504854	SIRT1 potent activator acts as anti-inflammatory	Phase II clinical trial on mild-to-moderate AD patients Dosage: 500 mg oral resveratrol q.d. Primary endpoint: Number of adverse events Change from baseline on volumetric magnetic resonance imaging (MRI) Secondary endpoint: ADCS-ADL CSF Aβ level	(Drygalski et al., 2018) (Moussa et al., 2017) (Turner et al., 2015)

Other potential treatments for AD include those based on the inhibition of glycogen synthase kinase-3β (GSK-3β) and mammalian target of rapamycin (mTOR) to attenuate neuroinflammation by increasing Aβ clearance and decreasing tau phosphorylation (Modrego and Lobo, 2019). Normally, extracellular ligands bind to a receptor tyrosine kinase (RTKs), which activate PI3K signaling cascades and lead to the activation of PDK-1 and Akt. PDK1 also indirectly activates the mTORC2 complex which activates Akt through the phosphorylation of the kinase at S473 and S450. Active Akt phosphorylates and activates tuberous sclerosis complex (TSC) 1 and 2, a negative regulator of mTORC1 (Hermida et al., 2017). mTOR inhibition activates the ubiquitin proteasome system and autophagy. In AD brain, where PI3K/Akt signaling pathway is downregulated due to Aβ plaques-induced neurotoxicity, Akt is suppressed and mTORC1 activity is increased, which disrupts cell autophagy, leading to neuroinflammation (Shal et al., 2018). Wang and colleagues reported rapamycin's (mTORC1 inhibitor) ability to inhibit elevated activation of mTORC1 and pro-inflammatory cytokines in the hippocampus of AD rats (Wang et al., 2016). Suppressed Akt also promotes activation of GSK-3β to cause hyperphosphorylation of tau that aggregates to form NFTs once detached from the microtubules. The loss of microtubules' integrity induces neuroinflammation and increases the risk of neuronal death (Mancinelli et al., 2017).

Methylene blue (MB) is one of the anti-tau disease modifying agents (DMTs) that combats tau pathology through two molecular targets: GSK-3β and tau aggregation (Gandini et al., 2018). Hyperphosphorylation of tau is associated with the loss of counterbalance between the kinases and phosphatases involved in tau phosphorylation, especially if the phosphorylation takes place at the sites of kinases as this family of enzymes regulate most of the protein function (Pradeepkiran et al., 2019). Kinases involved in tau phosphorylation are mitogen-activated protein kinases (MAPKs), cyclin-dependent kinases (Cdks), GSK-3β, and protein kinase A (PKA) (Kheiri et al., 2018; Li et al., 2018).

Initially, MB was well-known for its inhibiting activity on tau aggregation in AD clinical trials (Gureev et al., 2019). In spite of the advance in knowledge on tau pathology, partial inhibition on tau aggregation by MB is not adequate to halt AD as the underlying event that causes tau-mediated neurotoxicity is the binding of granular tau oligomers during NFT formation (Soeda et al., 2019).

Binding of Aβ plaques or glutamate to synaptic receptors can initiate the production of cyclic adenosine monophosphate (cAMP) from adenylyl cyclase, activating the cAMP/PKA pathway. Under normal conditions, downstream cAMP/PKA pathway results in phosphorylation of transcription factors such as cAMP response element binding protein (CREB) at S133, which stimulates transcription and translation of genes involved in the formation of memory (Bartolotti et al., 2016; Gao et al., 2018). However, the level of p-PKA was significantly decreased in the hippocampus of AD mice while neuroinflammation was found to be increased (Cai et al., 2018). Decreased PKA subsequently decreased the phosphorylated CREB in the rats' hippocampus (Huang et al., 2019). High levels of BACE1 in AD brain inhibits adenylyl cyclase and impairs cAMP/PKA pathway, which interrupts the phosphorylation and eventually disrupts the transcription and translation of CREB-induced genes, leading to memory impairment in AD (Chen et al., 2012). CREB activation can restore memory impairment in AD as the CREB-induced genes, such as brain-derived neurotrophic factor (BDNF) and insulin-like growth factor 1, can enhance neuron morphological outgrowth and formation of long-term and short-term memories (Kubota et al., 2017).

Metformin is a first-line medication for type 2 diabetes, which was repurposed for AD treatment as it exhibits anti-inflammatory properties and neuroprotective features against cognitive deterioration in AD (Ou et al., 2018). Metformin hinders the neuronal apoptosis and promotes neurogenesis in the hippocampus through the activation of the AMP-activated protein kinase (AMPK) pathways, which leads to the improvement of memory formation. AMPK can be phosphorylated by 3 key kinases, such as the liver kinase B1 (LKB1) complex at T172, due to increased cytoplasmic level of AMP, increased cytosolic Ca^2+^, and mitogen-activated protein kinase 7 (MAP3K7), also known as transforming growth factor beta-activated kinase 1 (TAK1) (Wang X. et al., 2019). AMPK also activates TSC1/2 complex that inhibits mTOR (Wang X. et al., 2019). Activity of AMPK was decreased in the hippocampus of AD rats at age 4-5 months, while the activity of mTOR increased, causing disrupted cell autophagy and exacerbated AD (Du et al., 2015; Sun et al., 2019). Inhibition of mTOR by rapamycin restores normal cell autophagy and protein synthesis (Sun et al., 2018).

PKA was found to activate silent information regulator type 1 (SIRT1), a neuroprotective protein deacetylase that reduces tau acetylation and downregulates BACE1, hence, increasing degradation of tau and reducing Aβ production (Zhang et al., 2019; Wang X. et al., 2019). Resveratrol, through activation of SIRT1, reverses the drastic decrease in hippocampal expression of SIRT1 in AD (Turner et al., 2015; Hou et al., 2017). Resveratrol was also found to reduce neuronal apoptosis and eventually restore cognitive impairment in AD (Tian et al., 2016).

Current DMTs target either Aβ pathology or tau pathology, which may be the reason for their lack of efficacy since both pathologies synergistically cause cognitive degeneration with the cholinergic deficit as a constant concern in AD. Multi target-directed ligand (MTDLs) is a novel approach to surmount the multifactorial AD pathogenesis (Geldenhuys and Darvesh, 2015). RPEL was synthesized through the combination of N, N'-disubstituted piperazine anti-amyloid scaffold and tacrine, into one compound (Sergeant et al., 2019). It was found effective in preventing cognitive impairment as it minimizes the Aβ plaques formation and tau phosphorylation in AD mice without any adverse effect besides maintaining the inhibitory activity on AChE. This approach accelerates the development of potential treatment for AD by minimizing the cost and time since the individual therapeutic effects of each compound is generally known (Hassan et al., 2019).

## Conclusion

Despite decades of research, we are still encountering a lack of success in pharmacotherapy of AD, mostly due to the multifactorial etiologies of the disorder that can initiate neurodegeneration interdependently. At present, combination therapy targeting several factors simultaneously appears to be promising. Additionally, an increasing number of studies are also focusing on neuroprotection against neuroinflammation. The impact of neuroinflammation interceded by microglia and astrocytes in AD pathogenesis is of great interest as it opens new doors for novel therapeutic targets. In addition to pharmacotherapy, better prognosis through early detection of AD biomarkers or brain imaging will enable early intervention that could potentially prevent the deposition of Aβ plaques and manifestations of various irreversible symptoms of AD.

## Author Contributions

NI and JK performed the literature search and drafted the manuscript. MY, WM, ST, and CH reviewed and finalized the manuscript.

## Funding

This study was supported by the GUP-2018-055. Fund provided by the National University of Malaysia (UKM).

## Conflict of Interest

CH was employed by Glyco Food Sdn Bhd.

The remaining authors declare that the research was conducted in the absence of any commercial or financial relationships that could be construed as a potential conflict of interest.
